# Restoration of enteroendocrine and pancreatic function after internal hernia and short bowel syndrome in a young woman with gastric bypass – a 2‐year follow‐up

**DOI:** 10.14814/phy2.13686

**Published:** 2018-05-06

**Authors:** Märta Borghede, Lars Vinter‐Jensen, Henrik H. Rasmussen, Simon Veedfald, Jens F. Rehfeld, Bolette Hartmann, Jens J. Holst, Filip K. Knop, David P. Sonne

**Affiliations:** ^1^ Center for Nutrition and Bowel Disease Aalborg University Hospital Aalborg Denmark; ^2^ Department of Biomedical Sciences Faculty of Health and Medical Sciences University of Copenhagen Copenhagen Denmark; ^3^ Novo Nordisk Foundation Center for Basic Metabolic Research Faculty of Health and Medical Sciences University of Copenhagen Copenhagen Denmark; ^4^ Department of Clinical Biochemistry Rigshospitalet, University of Copenhagen Copenhagen Denmark; ^5^ Steno Diabetes Center Copenhagen for Diabetes Research Gentofte Hospital University of Copenhagen Hellerup Denmark; ^6^ Department of Clinical Medicine Faculty of Health and Medical Sciences University of Copenhagen Copenhagen Denmark; ^7^ Department of Clinical Pharmacology Bispebjerg and Frederiksberg University Hospital Copenhagen Denmark

**Keywords:** Gastric bypass, gut hormones, RYGB, short bowel syndrome

## Abstract

A serious complication to the laparoscopic Roux‐en‐Y gastric bypass (RYGB) is internal hernia, which can lead to massive bowel necrosis that may result in short bowel syndrome. We determined postprandial enteropancreatic hormonal responses and metabolites in a 22‐year‐old nondiabetic woman with a history of RYGB experiencing severe internal herniation with widespread bowel necrosis. Extensive resections were performed leaving her with a saliva fistula from the pouch‐enteric anastomosis, an intact duodenum, 15 cm of jejunum, 35 cm of ileum, and intact colon. Parenteral nutrition was initiated and 10 months after the bowel resection, intestinal continuity was re‐established. After 6 weeks the patient reached parenteral nutrition independence. She underwent standardized liquid mixed meal tests before, 3 months after and 2 years after intestinal continuity was re‐established. Gut hormone responses were completely restored postoperatively leading to very high concentrations in plasma. After 2 years, plasma concentrations had, however, decreased markedly, suggesting desensitization of the gut ostensibly in response to chronic hyperstimulation. There was no evidence of cephalic phase insulin secretion.

## Introduction

The gastrointestinal architecture after Roux‐en‐Y gastric bypass (RYGB) surgery elicits increases in the exposure of distal parts of the small intestine to nutrients while the enteroendocrine cells in the duodenum are excluded from the nutrient flow. Enteroendocrine L cells in the distal parts of the small intestine respond with increased secretion of glucagon‐like peptide‐1 (GLP‐1), glucagon‐like peptide‐2 (GLP‐2) and peptide YY (PYY). GLP‐1 and glucose‐dependent insulinotropic polypeptide (GIP), which are secreted from K cells in the upper small intestine, stimulate the secretion of insulin (incretin effect) (Holst 2013). Moreover, GLP‐1 and PYY, together with cholecystokinin (CCK) from the upper intestine, inhibit appetite, gastric emptying and food intake. GLP‐1 in addition suppresses glucagon secretion, which together with GLP‐1‐induced deceleration of gastric emptying and GLP‐1‐induced insulin secretion is crucial for the regulation of postprandial glucose homeostasis. GLP‐2 exerts trophic effects on the intestinal mucosa (Drucker 2001). GLP‐2 also enhances the absorption of nutrients and gut adaptation in patients with various resections. RYGB results in favorable effects on glucose and energy homeostasis, but can also be associated with severe complications resulting in intestinal failure, hypoglycemia, and short bowel syndrome (Borghede et al. 2013).

**Figure 1:**
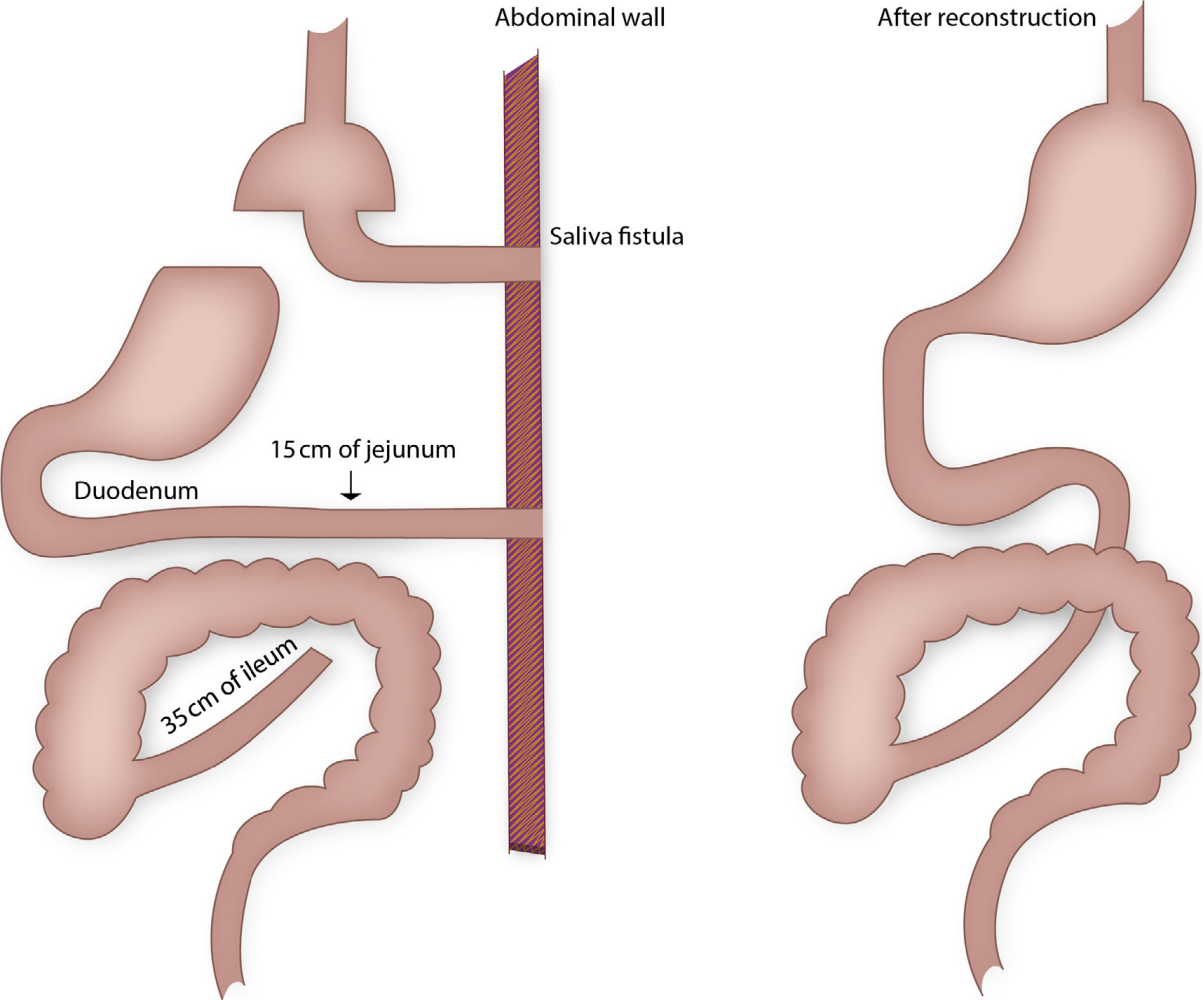
The intestinal system of a 22‐year‐old woman, with a history of Roux‐en‐Y gastric bypass, complicated with internal hernia and massive bowel necrosis. After extensive bowel resection the patient ended up with a saliva fistula from the pouch‐enteric anastomosis, a jejunostomy 15 cm from the ligament of Treitz, and a blind closed ileum 35 cm from the ileo‐coecal valve (left). Ten months later the intestinal continuity was re‐established (right).

We determined metabolites and postprandial gastrointestinal and pancreatic hormone concentrations in plasma and serum from a 22‐year‐old nondiabetic woman who had experienced severe internal herniation after RYGB and subsequent extensive bowel resection resulting in short bowel syndrome (Fig. 1) (Borghede et al. 2013).

## Materials and Methods

The patient received a standardized (800 kcal) liquid test meal (90 g carbohydrate, 31 g fat, 40 g protein) 7 days before, 3 months after and 2 years after restoration of gastrointestinal continuity. Three resections had left the patient with a saliva fistula 10 cm below the pouch‐enteric anastomosis, a jejunostomy 15 cm from the ligament of Treitz and a blind closed ileum 35 cm from the ileo‐coecal valve. 10 months after the intestinal continuity was reestablished (Fig. 1) (Borghede et al. 2013). The patient had her gallbladder removed prior to herniation, but was otherwise healthy. Prior to surgical reestablishment of intestinal continuity, she received parenteral nutrition 4 days a week, which was discontinued 6 weeks after surgery. Paracetamol was added to the last liquid meal for evaluation of gastric emptying. Each meal test was performed after an overnight fast (10 h). The patient was positioned in a 45° recumbent position and had a cannula inserted in the cubital vein. Blood was arterialized by wrapping the lower arm and hand in a heating pad. Blood samples were drawn repeatedly and distributed into chilled EDTA tubes prepared with Trasylol (Nordic Drugs, aprotinin, 500KIU/mL) for plasma analysis of gastrointestinal hormones and metabolites. For measurements of insulin and C‐peptide, blood was distributed into chilled tubes containing heparin. Tubes were kept on ice until centrifugation (20 min at 1200 g, 4°C). Plasma for the analysis of glucagon, GIP, GLP‐1, GLP‐2, polypeptide (PYY), CCK, gastrin, pancreatic polypeptide (PP), and somatostatin was stored at −20°C and plasma samples for insulin and C‐peptide were stored at −80°C. For the analyses of paracetamol and bile acids blood was distributed into dry tubes for coagulation (20 min at room temperature). For the analyses of plasma glucose, blood was collected in sodium‐fluoride‐coated tubes and centrifuged immediately at 7400 *g* for 2 min at room temperature. Glucose concentrations were measured bedside by the glucose oxidase method (Yellow Springs Instrument model 2300 STAT plus analyzer; YSI, Yellow Springs, OH, USA). Plasma insulin, C‐peptide and triglyceride concentrations were measured using a two‐sided electrocheminescence immunoassay (Roche/Hitachi Modular analytics; Roche Diagnostics, Mannheim, Germany). Gastrointestinal hormones were measured by radioimmunoassays as described elsewhere (Sonne et al. 2014). Serum paracetamol was measured by the Vitros ACET slide (Sonne et al. 2014). Total serum bile acids were measured using the enzymatic colorimetric method (Konelab 30i, Thermo Fisher Scientific, Hvidovre, Denmark).

## Results

### Weight

The patients’ weight was 54 kg before the operation, 57 kg at 3 months and 76 kg after 2 years. The corresponding body mass indices were 19.7 kg/m^2^, 20.7 kg/m^2^, and 27.6 kg/m^2^, respectively.

### Glucose, insulin, C‐peptide and glucagon

**Figure 2:**
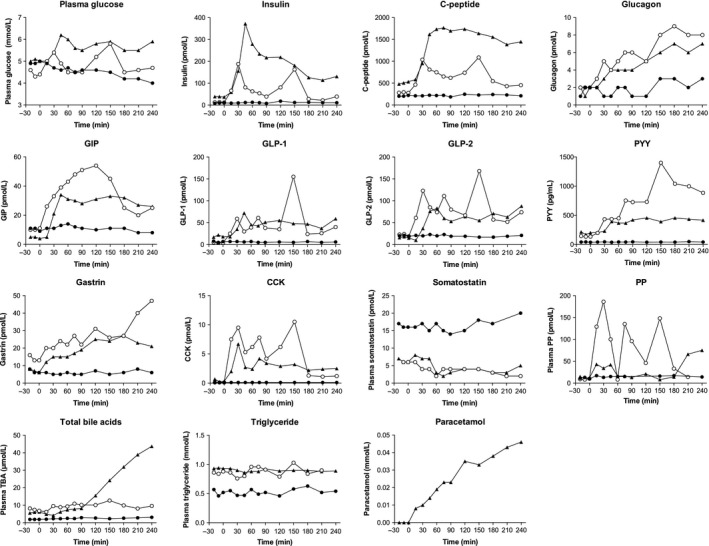
Postprandial plasma/serum concentrations of glucose, insulin, C‐peptide, glucagon, glucose‐dependent insulinotropic polypeptide (GIP), glucagon‐like peptide‐1 (GLP‐1), glucagon‐like peptide‐2 (GLP‐2), peptide YY (PYY), gastrin, cholecystokinin (CCK), somatostatin, pancreatic polypeptide (PP), total bile acids (TBA), triglycerides and paracetamol before (solid circles), 3 months after (open circles) and 2 years after (solid triangles) intestinal continuity was re‐established.

Before surgery, plasma concentrations of glucose, insulin, C‐peptide and glucagon remained at baseline throughout the meal test (Fig. 2). Three months after reconstructive surgery, postprandial glucose, insulin and C‐peptide concentrations exhibited bi‐phasic profiles with peaks at 30 min and 150 min. After 2 years, postprandial glucose, insulin and C‐peptide concentrations were markedly increased. Peak concentrations of both glucose, insulin and C‐peptide appeared early (~45 min) with C‐peptide plateauing at 1500–1700 pmol/L, whereas insulin concentrations decreased after peaking at 370 pmol/L. Glucose concentrations plateaued at 5.5–6.2 mmol/L after ~30 min. Baseline concentrations of glucagon were low (1–2 pmol/L) before and after surgery. After surgery, postprandial concentrations increased slowly and peaked after 180 min (7–8 pmol/L), with slightly higher concentrations at 3 months compared to 2 years postoperatively.

### GIP, GLP‐1, GLP‐2, and PYY

Three months postoperatively, GIP increased markedly between 30 and 120 min reaching peak concentrations at ~50 pmol/L (Fig. 2). After 2 years, GIP concentrations peaked at 45 min (~30 pmol/L) and remained somewhat constant throughout the test. Two months postoperatively, GLP‐1 and GLP‐2 concentrations reached 50 pmol/L and 100 pmol/L, respectively, and both hormones exhibited high peak concentrations (~150 pmol/L) at 150 min. At the two‐year follow‐up, no late peaks were seen and all three hormones remained at their respective plateaus (GIP ~50 pmol/L, GLP‐1 ~50 pmol/L, GLP‐2 ~60‐70 pmol/L). Similarly, PYY concentrations increased progressively reaching 1400 pg/mL at 150 min 3 months postoperatively. After 2 years, PYY concentrations reached ~500 pg/mL after 45 min and remained here throughout the meal test.

### CCK, gastrin, somatostatin, and total bile acids

CCK concentrations exhibited three peaks (8‐10 pmol/L) at the three‐month follow‐up (Fig. 2). The last and largest peak was seen at 150 min (~10.5 pmol/L). After 2 years, this pattern remained, but at lower concentrations. Gastrin concentrations increased throughout the meal tests reaching peak concentrations of 27 pmol/L (3 months) and 47 pmol/L (2 years), respectively. Somatostatin concentrations remained constant at 15–20 pmol/L before the operation. After reconstructive surgery, basal concentrations decreased to ~6 pmol/L and exhibited postprandial suppression at 60 min. Total bile acid concentrations after 3 months reached 10–12 *μ*mol/L and returned to baseline after 3–4 h. However, 2 years after surgery, bile acid concentrations increased throughout the meal test reaching 44 *μ*mol/L at 240 min.

### PP, triglyceride and paracetamol

PP remained at baseline in the preoperative setting (Fig. 2). Three months after reconstructive surgery three large PP peaks (~140–180 pmol/L) were observed at 30, 75, and 150 min, respectively. After 2 years, however, only a minor increase in PP was observed at ~15 min and again at ~180 min. Triglyceride concentrations showed no postprandial changes before or after surgery, but concentrations increased from 0.5 mmol/L before surgery to ~1 mmol/L after reconstruction. Paracetamol concentrations (a surrogate marker of gastric emptying) increased throughout the meal test indicating gradual, regulated emptying of the gastric contents of the meal.

## Discussion

We present enteroendocrine and pancreatic endocrine responses in a young woman with severe complications after RYGB resulting in short bowel syndrome. A key finding of the study is the demonstration of the ability of the gut to adapt and restore both its pancreatic and endocrine function after having remained dormant for more than 10 months. Meal tests were carried out before and after restoration of intestinal continuity. Postprandial plasma concentrations of the gastrointestinal and pancreatic hormones were unaffected by food intake prior to the re‐establishment of intestinal continuity, but were restored after re‐establishment of intestinal continuity. Because of the unique anatomy prior to reconstructive surgery, the meal may be considered a true sham feeding.

### Glucose regulation, L cell products, and gastric emptying

Glucose as well as insulin/C‐peptide concentrations increased during the 2 years. These changes may represent the return of an insulin resistant state because of the patient′s weight gain. Two years after reconstructive surgery, postprandial concentrations of GLP‐1, GLP‐2, PYY, and GIP were blunted compared to the marked release observed at 3 months. This suggests that the gut becomes desensitized when hyperstimulated over time. A similar ‘adaptive pattern’ was also observed for CCK and gastrin. However, based on paracetamol absorption and GIP concentrations (known to reflect the gastric emptying), an element of gastric retention also seems evident. Indeed, even gastrin, which is secreted from the antrum of the stomach, continued to increase during the postoperative meal tests. Clinically, however, the patient has never presented symptoms of gastric retention. Neither did imaging of the stomach and intestine with contrast show any signs of retention or stenosis at the level of the anastomoses.

The early postprandial increase of GLP‐1, GIP, and GLP‐2 concentrations were comparable to those observed after RYGB surgery (Jacobsen et al. 2012; Jørgensen et al. 2012), but our patient also exhibited a high peak after 150 min, indicating a possible stimulation of colonic L cells. Normally, this peak is not observed during mixed meal tests performed in healthy subjects or in RYGB patients (Jacobsen et al. 2012; Jørgensen et al. 2012). Neither did it appear during the meal test at two‐year follow‐up. Likewise, PYY, a satiety hormone at least partially co‐secreted with GLP‐1, reached a peak concentration of 1400 pg/mL (~330 pmol/L) at 150 min ‐ a considerably higher peak than normally observed following RYGB surgery (Korner et al. 2005). GLP‐2 exerts trophic effects on the intestine, enhancing absorption of nutrients and promoting intestinal growth, which is beneficial for patients with short bowel syndrome (Drucker 2001). Indeed, 6 weeks after the intestinal continuity was re‐established our patient was able to manage without parenteral nutrition (Borghede et al. 2013). The re‐established intestinal continuity and an intact colon are important for the restoration of L cell secretion, and our data therefore suggest that the colon may substitute for the small intestine with regards to the secretion of these hormones. Indeed, enhanced L cell secretion might facilitate increased absorption capacity of the small intestine and even promote intestinal growth (Jeppesen et al. 2012; Hvistendahl et al. 2016). Basal glucagon concentrations were low and postprandial concentrations increased progressively during both postsurgery meal tests. Hyperglucagonemia was not present and glucagon concentrations stayed below 10 pmol/L.

### Cephalic insulin secretion and PP

Because of the anatomical situation before surgery, the meal test represents sham feeding. However, PP concentrations before reconstruction were unaffected by meal intake, which suggests lack of vagal signaling (probably as a consequence of the operations). PP, however, increased postoperatively probably reflecting intestinal stimulation. Accordingly, there was no sign of cephalic phase insulin secretion, which is thought to take place during the first 10 minutes following meal intake (Teff et al. 1991; Ahrén and Holst 2001), although findings in humans are inconsistent as some studies do not find cephalic phase insulin secretion (Crystal and Teff 2006; Veedfald et al. 2016). A cephalic insulin response could also have gone unnoticed due to the study design, in which a 15‐min sampling interval was applied.

### Gastrin, CCK, somatostatin, bile acids, and triglycerides

Postprandial plasma concentrations of CCK were comparable to those normally seen after RYGB and a clear reduction was observed 2 years after surgery (Jacobsen et al. 2012). Three CCK peaks were observed which could represent variable CCK processing or secretion throughout the intestine. Gastrin concentrations increased during the entire meal test, which could indicate a gradual emptying of the gastric contents postoperatively – as also suggested by the paracetamol/GIP data. We observed reduced somatostatin (measured in a way to include both the 1–14 and the 1–28 forms) secretion after the operation, which is surprising since some of the somatostatin cells in the intestinal mucosa are of the open type and would be expected to respond to luminal stimulation. Together with the finding of suppressed postprandial somatostatin concentrations, these results could indicate that stimulation of the gut per se impacts negatively on somatostatin release. Total bile acid concentrations increased massively at the two‐year follow‐up confirming previous findings of high bile acid concentrations years after RYGB surgery (Jørgensen et al. 2015). Most likely, gut adaptation or remodeling resulting in increased transport of both conjugated and unconjugated bile acids is a likely explanation. Triglycerides were unaffected by this increase in bile acid transport and postoperative plasma concentrations did not indicate improved absorption over time.

In conclusion, our patient experienced severe internal herniation after RYGB surgery and underwent extensive bowel resections resulting in short bowel syndrome and disappearance of normal postprandial enteroendocrine and pancreatic endocrine responses. The meal test performed prior to reconstructive surgery was interpreted as a human sham‐feeding test, but no cephalic phase release of insulin was demonstrated. Three months after reconstructive surgery postprandial gut hormone concentrations were fully restored and maintained for 2 years, albeit at lower concentrations with time. Glucose, insulin/C‐peptide and weight, however, increased postoperatively and total bile acid concentrations were also markedly increased 2 years after surgery.

## Conflict of Interest

All authors have reported no relevant conflicts of interest.

## Patient consent

Obtained.
